# Variation in the Commissioning of Semaglutide for the Treatment of Obesity and Overweight Across England: Results of Three Freedom of Information‐Based Mapping Exercises Across the 42 Integrated Care Boards of England

**DOI:** 10.1111/cob.70044

**Published:** 2025-09-11

**Authors:** Anne de Bray, Hanna Schnitzer, Elisabeth Mahase, Puspha Singh, Rob Andrews, Barbara M. McGowan, Sarah Le Brocq, John P. H. Wilding, Stuart W. Flint, Jonathan M. Hazlehurst

**Affiliations:** ^1^ Institute of Metabolism and Systems Science University of Birmingham Birmingham UK; ^2^ Oxford Centre for Diabetes, Endocrinology and Metabolism (OCDEM) Oxford UK; ^3^ Sky News Ltd London UK; ^4^ The British Medical Journal (BMJ) London UK; ^5^ Institute of Applied Health Research University of Birmingham Birmingham UK; ^6^ Department of Diabetes and Endocrinology University Hospitals Birmingham NHS Foundation Trust Birmingham UK; ^7^ Centre for Endocrinology, Diabetes and Metabolism, Birmingham Health Partners Birmingham UK; ^8^ Medical School University of Exeter Exeter UK; ^9^ Endocrinology, Guy's and St Thomas' Hospitals NHS Trust London UK; ^10^ Reset Health, Fleet Place London UK; ^11^ Department of Cardiovascular and Metabolic Medicine, Institute of Life Course and Medical Sciences University of Liverpool Liverpool UK; ^12^ School of Psychology University of Leeds Leeds UK; ^13^ Scaled Insights, Nexus, University of Leeds Leeds UK

**Keywords:** access, commissioning, obesity, treatment

## Abstract

Obesity medications are recommended in England with legislation necessitating their availability. However, given the number of people who meet clinically approved eligibility criteria, funding these medications and associated support services may limit efficacy at a population health level. This study aimed to assess the commissioning and availability of services and obesity medications across England. Three sets of freedom of information requests were sent to the 42 ICBs in England by Sky News Ltd, The BMJ and the study investigators of this work with questions focused on commissioning of services and medication eligibility and prescription across England. The three data sets were combined to provide a narrative description to inform further development in obesity care. The availability of services across England was partial, and when services did exist, medication access was limited by funding and more restrictive eligibility criteria beyond those approved by the National Institute of Health and Care Excellence. Subsequently, very few patients receive NHS prescriptions even in areas where funding medications are reportedly available. The capacity of services to offer comprehensive care for patients to receive obesity medications is insufficient to meet current demand. Despite legislation for the delivery of obesity medications, these treatment options are not widely available on the NHS. There is insufficient service capacity to provide comprehensive care for eligible patients seeking obesity medications as a treatment option.

**Summary**

- What is already known about this subject
  ○ Obesity medications can be an effective treatment option
  ○ Obesity medications are to be used by services able to provide comprehensive wraparound support.
  ○ The availability of these services and their funding may limit access to treatment.
- What this study adds
  ○ There is not enough capacity in specialist services to treat all patients seeking treatment.
  ○ There is considerable variation in the funding of services and obesity medications.
  ○ Even when obesity medications are reportedly funded, very few patients are prescribed these treatments within England.

## 1 Introduction

Obesity medications in the form of Nutrient‐Sensing Hormone‐based therapies (NuSH) have the potential to significantly improve health outcomes for patients living with obesity. Equitable and widespread access to treatment‐funded pathways are required, not only to the medications but also the associated wraparound support in the form of supported self‐management approaches for diet, physical activity, and psychology. Within England, the National Institute for Health and Care Excellence (NICE) has assessed the available medications in this category for their use in patients living with overweight or obesity, which has resulted in three technology appraisals (TAs) for liraglutide (Saxenda) [1] semaglutide (Wegovy) [2] and most recently tirzepatide (Mounjaro) [3].

Integrated Care Boards (ICBs) are the NHS organisations involved in the planning of health services and commissioning of NHS budgets at a regional level, and they bear responsibility for delivering regional access to NICE‐recommended treatments or technologies [4]. Health systems are required to comply with the recommendations of NICE TAs within 3 months of publication or commercial availability of the technology [5]. The delivery of the liraglutide TA in particular was made more difficult due to the poor availability of the specialist services, called Tier 3 weight management services [6] that were required by the TA for it to be prescribed [1]. The TA for semaglutide reflected this challenge and softened the requirement of the type of service required for its prescription from “Tier 3 weight management services” to “Specialist Services” presumably in the hope of widening access [2]. Therefore, ICBs were obligated to provide access to semaglutide as an obesity treatment by June 2023 for patients eligible under the TA. However, despite this obligation, patients living with obesity still reported that they were unable to access semaglutide without cost on the National Health Service (NHS). This came at a time of increasing pressure on obesity services due to inadequate funding in the context of increased demand resulting from the perceived availability and efficacy of obesity medications and the associated media coverage [7, 8]. Professional societies, reflecting the challenge in delivering the semaglutide TA as well as concerns about cost and medication availability, recommended a phased introduction of obesity medications [9]. The full implementation of the semaglutide TA was further limited by concerns about its supply, which resulted in a medicines shortage notification (MSN) within the UK in July 2023 [10].

More recently, tirzepatide has been assessed by NICE, receiving a NICE TA [3]. Tirzepatide is to be made available to far more patients living with obesity through its eventual use in primary care rather than specialist services [3]. Whilst this is likely to lead to significant pressure on primary care providers, this may widen access to effective treatment in a more equitable way than reliance on the patchy provision of specialist services. As a result, there is increased interest in whether digitally delivered support alongside these medications is safe and effective [11]. Current digital NHS approved providers are intensive and more closely match traditional NHS Tier 3 weight management services, and in their current form, may not be able to meet the current demand. Given the increased interest and launch of tirzepatide, it is salient to reflect on the recent experiences with semaglutide to avoid the same mistakes and to take steps to address the inequity experienced by patients living with obesity who seek obesity medications and for services that are unable to provide comprehensive care in line with NICE TAs for obesity medications.

Previous government attempts to map provision of specialist weight management services have been limited by poor response rates from local authorities and clinical commissioning groups (the predecessor to the current ICBs), impacting the quality of data collected and representation across England [12]. To overcome this limitation, we leveraged the power of the Freedom of Information (FOI) Act (2000) which legally requires institutions to respond to a request for information within 20 working days of receipt [13].

At the time of ongoing work from the Obesity Management Collaborative (OMC), UK using FOI to map semaglutide provision, the BMJ [14] and Sky News Ltd [15] published findings from their own FOI requests. Thus, three FOI requests were distributed across England simultaneously, requesting information relating to the delivery of obesity medications. Both The BMJ and Sky News Ltd agreed to contribute their FOI data sets to the work by the OMC to provide an accurate and detailed description of the delivery of obesity medications in clinical care across England.

## 2 Methods

FOI requests were sent by three separate groups working on behalf of different organisations: The BMJ, Sky News Ltd and the Obesity Management Collaborative (OMC). The three sets of FOI requests had considerable overlap as well as some key differences pertinent to this and future research involving FOI requests.

*The BMJ*: Author E.M. is a journalist for The BMJ's news. E.M. submitted the questions in Table 1 under the FOIA to the 42 ICBs in England on 24.07.24.

**TABLE 1:** FOI questions submitted to Integrated Care Boards (ICBs).

The BMJ	Sky News Ltd.	Obesity Management Collaborative (OMC)
Do you commission a Tier 3 specialist weight management service? Is it located within your ICS catchment? If not, where are people within your ICS requiring Tier 3 weight management services typically referred? If you commission a tier 3 service, is it currently accepting new referrals? And if it is not accepting new referrals, when did this come into effect? Is there a Wegovy pilot site located within your ICS?	Do you refer patients to a Tier 3 weight management service (WMS)? If yes, how many patients are currently in your Tier 3 WMS? How many patients were on the waiting list for Tier 3 WMS as of 30/04/24? What is the average waiting time for people to be seen by the service as of 30/04/24? Do you offer Wegovy? If yes, please specify any other eligibility criteria you impose in addition to the NICE technology appraisal How many patients received Wegovy between 01/09/2023–30/04/2024? How many patients do you have capacity/resources to treat annually with Wegovy?	Does your ICB currently fund prescription of Semaglutide 2.4 mg (Wegovy) within specialist weight management services? Please name the specialist weight management services you commission If you do not commission a specialist weight management service, please name any specialist weight management services that operate within your ICB geography that you are aware of and are not commissioned? Are there any additional criteria used to restrict eligibility in your ICB for Semaglutide 2.4 mg outside of the recommendations in NICE TA875? Since 8th March 2023, how many letters of complaint has your ICB received from patients who feel they should receive Semaglutide 2.4 mg? If you do not currently fund Semaglutide 2.4 mg is the ICB working to implement this? If you are looking to implement Semaglutide 2.4 mg is it projected to be funded by your ICB before April 2025?

*Note*: Questions regarding the provision of Tier 4 weight management services and bariatric surgery were also submitted by Sky News Ltd. and The BMJ but are presently omitted as it is beyond the scope of discussion for this study. Several ICBs directed the authors to contact their local NHS Trusts to obtain the information. Several ICBs signposted to publicly available information, such as regional formularies or statements on Semaglutide provision, as part of their response in accordance with Section 21 of the FOIA. Where responses were unclear, further clarification was sought.

*Sky News Ltd*: Author H.S. is a specialist producer at Sky News Ltd. H.S. sent FOI requests as part of an investigation for their employer, Sky News Ltd. H.S. submitted the questions in Table 1 to ICBs (23.05.24–08.07.24) and 109 specialist weight management providers (23.05.24–29.07.24) in England.

*Obesity Management Collaboration*: On behalf of the OMC, authors A.d.B., J.P.H.W., J.M.H. and R.A. drafted seven questions to be included in the FOI requests to ICBs, with further review and refinement by B.M.M. and S.L.B. The FOI were sent to 42 ICBs on 18–19.07.24.

## 3 Results

*The BMJ*: all 42 ICBs responded to the majority of questions. Three ICBs did not provide an answer to question 2, whilst two ICBs did not provide an answer for question 3.

*Sky News Ltd*: responses to all questions were provided by 39/42 ICBs or the NHS trusts that the authors were referred to (29/42 ICBs stated that we were unable to answer the questions and referred the authors to local NHS Trusts to respond). Partial information was acquired for 2/42 ICBs; outstanding information has still not been received since July 2024. No information was obtained from one ICB as the authors were referred to an NHS Trust, which has not provided information since submitting the FOI in June 2024, with no response to ongoing follow‐up enquiry.

*OMC*: all 42 ICBs responded to the OMC; one ICB stated that it did not hold the relevant information to provide a response. This same ICB also referred the Sky News Ltd authors to approach their acute NHS Trust, which responded to the BMJ FOI request informing that they do not currently fund a Tier 3 weight management service.

### 3.1 Commissioning and Prescription of Semaglutide

#### 3.1.1 Commissioning of Semaglutide

There were differences in the number of ICBs which report commissioning of semaglutide between data sets. It is important to note that (1) the majority of ICBs stated to Sky News Ltd that they did not hold the information so the authors were required to submit further FOI or press requests to local NHS Trusts and other providers, and (2) Sky News Ltd FOI question asked if providers “offer” semaglutide, whilst The BMJ and OMC FOI request asked if semaglutide is commissioned in their ICB area. Thus, FOI team responses and our interpretation could vary. For example, the ICB may commission semaglutide but the local NHS Trust may not currently offer it if it does not have a specialist weight management service or they are unable to obtain the medication, so the response for the ICB would be noted as “not offering.” The results summarised for each ICB, which re‐directed the FOI request to local NHS Trusts or providers, may therefore have a high chance of being incomplete or inaccurate regarding commissioning.

In response to Sky News Ltd, 48% (19/40) of ICBs (or their designated local NHS Trusts/providers) reported that they do offer semaglutide, whilst 50% (20/40) ICBs do not offer semaglutide and one stated that patients can access it outside of their ICB. Two ICBs did not respond to the FOI request.

However, the OMC received responses directly from 41/42 ICBs to the question ‘1. Does your ICB currently fund prescription of semaglutide 2.4mg within specialist weight management services?’ Given the specificity of the question and the response directly from the ICB, rather than delegated to local NHS Trusts/providers, the authors feel that the data gained from this question is likely to be more reflective of the true state of semaglutide commissioning. The responses to OMC FOI requests found that (85%) 35/41 ICBs currently report commissioning semaglutide and 15% (6/41) do not.

The BMJ FOI identified that 2/39 responding ICBs reported that they were semaglutide pilot sites, 36/39 were not, and one ICB gave an unclear answer. Two ICBs did not respond.

#### 3.1.2 Eligibility Criteria for Semaglutide Prescription

Sky News Ltd received information about 40/42 ICBs to their question ‘please specify any other eligibility criteria you impose in addition to the NICE technology appraisal’. They found that 12.5% (5/40) ICBs report that they do not have any additional eligibility criteria beyond NICE TA875 to restrict access to semaglutide. 17.5% (7/40) are prescribing in a phased approach, with prescribing is limited to those in Phase 1. 2/42 ICBs stated that they only prescribe semaglutide for instances of life‐threatening obesity or for those on a Tier 4 surgical waiting list. 60% (25/42) ICBs responded with ‘not applicable’; 20/40 ICBs were found to not be offering semaglutide.

The OMC received very different data. Forty‐one ICBs responded directly but one ICB's response was unclear. The OMC found that 78% (31/40) ICBs do not have any additional eligibility criteria for the prescription of semaglutide above NICE TA875. However, 4/31 currently prescribe only for those in Stage 1 of a phased approach and one service is closed to new referrals. Therefore, 65% (26/40) ICBs are reportedly fully compliant with NICE TA875. A further 2/40 have additional eligibility criteria which restrict access to semaglutide.

#### 3.1.3 Data Regarding Semaglutide Prescription

In response to the question ‘How many patients received Wegovy between 01/09/2023–30/04/2024?’, Sky News Ltd found that across 40 responding ICBs, 838 patients were prescribed semaglutide. This is an average of 44 people (mean, range 0–331, median 22) within each ICB.

Of the five ICBs that had prescribed no patients with semaglutide, three ICBs (or their local NHS Trust) stated that their policy for prescribing was agreed after the time frame in question; one stated that no stock was available, and one gave no justification.

In response to the question ‘How many patients do you have capacity/resources to treat annually with semaglutide?’ across 40 responding ICBs, there was a reported capacity to prescribe for 2977 patients; an average of 213 (range 7–500) people per ICB.

#### 3.1.4 Complaints Regarding Access

Some ICBs reported numbers of enquiries as well as numbers of complaints; only complaints have been included for analysis. Some ICBs declined to give an exact figure, or stated that the figure was fewer than 5, to avoid the risk of identifying an individual, which is permissible under the Freedom of Information Act 2000, Section 40(2) (personal information).

The range of number of complaints was 0–42, median number of complaints was 0.5. 45% (18/40) of ICBs report they received no complaints, 35% (14/40) received 5 or less complaints and 20% (8/40) of ICBs received > 5 complaints.

#### 3.1.5 Future Plans to Commission Semaglutide

The OMC found that of the nine ICBs that are currently unable to prescribe semaglutide (either due to not funding semaglutide or a suitable weight management service), all are working to provide access to semaglutide. However, only 3/9 ICBs stated that their plans aimed to provide access to semaglutide by April 2025, with the 6/9 remaining ICBs stating that they were unable to provide a timescale.

### 3.2 Tier 3 Weight Management Service Funding and Provision

#### 3.2.1 Commissioning of Specialist Weight Management Services

The ICB responses to questions from the three groups of requesters regarding the current commissioning of specialist weight management services within their catchment area varied (Table 2). The BMJ received responses from all 42 ICBs, with 76% (32/42) reporting commissioning services that are located within their catchment area. 9.5% (4/42) ICBs stated that they have services which are accessible to part of their catchment area, with 12% (5/42) stating that they do not. One ICB states that they do not commission a specialist weight management service, but there are ‘some elements’ provided by their local NHS Trusts.

**TABLE 2:** ICB responses.

	Question regarding commissioning of weight management service	ICB responses
The BMJ	Do you commission a Tier 3 specialist weight management service? Is it located within your ICS catchment? If not, where are people within your ICS requiring Tier 3 weight management services typically referred?	42 responses: 32 Yes 4 Yes, partial cover 1 within Trusts^a^ 5 No
Sky News Ltd	Do you refer patients to a Tier 3 weight management service (WMS)?	41 responses: 33 Yes within ICB area 5 Yes, but only out of area services available 3 No
OMC	Please name the specialist weight management services you commission	41 responses: 36 Yes 5 No

*Note*: ICB responses related to questions regarding commissioning of specialist weight management services.

^a^ 1 ICB stated that it does not commission a specialist weight management service itself but some elements of a WM service in the NHS trusts that it funds.

However, a caveat to this data is that The BMJ question does not ask specifically if the services within their catchment area cover their entire population, so there may be more partial cover than stated.

Sky News Ltd received 41 responses, with 80% (33/41) referring patients to a service within their catchment area, 12% (5/41) referring patients to services outside of their catchment area, and 7% (3/41) without a commissioned specialist weight management service.

The OMC received 41 responses; the ICB which did not respond was different to the non‐responder to Sky News Ltd. OMC FOI requests found that 88% (36/41) of responding ICBs do commission specialist weight management services, matching The BMJ data that 12% (5/42) do not commission a service. However, of note, for one ICB, their sole commissioned service is non‐prescribing, meaning that semaglutide cannot be prescribed in that ICB region, one service is closed to new referrals (so only a maximum of 34/41 ICBs would appear to have a weight management service capable of prescribing semaglutide to new patients) and one service is a 3‐year trial due to end in 2026.

From OMC data, of the five ICBs that do not commission specialist WM services, two were aware of other providers in their region (one ICB stated that eligible patients are referred to these other providers, the other ICB referred to services commissioned by a neighbouring ICB but this service is currently not prescribing semaglutide pending a review), two were unaware of other weight management services within their region, and one did not hold the information.

#### 3.2.2 Current State of Specialist Weight Management Services

29/42 ICBs currently have WM services accepting referrals (The BMJ data) with 3/42 ICBs declaring that there is partial suspension of accepting new referrals in some services within their catchment area. 3/42 ICBs state that all services in their area have stopped accepting new referrals (The BMJ data).

To assess the number of patients within, and on the waiting list for, specialist weight management services, both ICBs and local providers were contacted, Sky News Ltd. The total number of patients reported to be within a Tier 3 weight management service is 51,661, with an average of 1614 (range 50–6956) from the 33 ICBs that provided information.

The total number of patients on waiting lists for Tier 3 weight management services was 49,690, with an average of 1734 (range 0–7154) from the 30 responding ICBs.

The average waiting time from referral to assessment in a Tier 3 weight management service is 7.9 months (median, range 2–36 months, IQR 7.75); this information was provided by 27/42 ICBs (Sky News Ltd).

The missing data is due to some ICBs unable to provide information themselves because they do not commission services, do not have access to the data locally or the services that their patients are referred to are out of their catchment area. Further, it is worth noting that data from each ICB was not always complete as no private providers provided any data [11].

#### 3.2.3 Profile of Providers of Specialist Weight Management Services

Across the 36 ICBs which report commissioning specialist weight management services, a total of 72 different services are commissioned, comprised of 54 (75%) NHS providers and 18 (25%) private providers (OMC data).

Most (64%, 23/36) ICBs commission solely NHS providers, with the rest commissioning a mix of NHS and private providers (25%, 9/36) or solely private providers (11%, 4/36) (OMC data).

As mentioned above, Sky News Ltd also contacted 109 potential providers of specialist weight management services either by ascertaining which NHS trusts work within the remit of the ICB or from deferral to the provider when ICBs stated that they could not provide the information. Providers contacted include acute NHS trusts, community NHS trusts, local Borough or County Councils, and private companies. 16/109 did not respond as they either did not hold the information or were private providers and so did not respond. 1/109 only provided partial information. 2/109 providers were mental health Trusts, so the request was not applicable to their service.

## 4 Discussion

This is the first published study to map commissioning of semaglutide across the 42 ICBs in England, using FOI requests. Interestingly, responses to similar questions were handled differently by ICBs. For Sky News Ltd, the majority of FOI requests were returned with a response for authors to contact individual NHS trusts or weight management service providers, whereas very similar questions by The BMJ or OMC were provided with direct responses from the ICB.

Given the similar timeframe (within 3–4 months) of FOI requests submissions from the three parties, it is unlikely that the variation in responses is due to changes in service provision but due to slight differences in wording affecting the data gathered by the ICB‐based FOI officers. Although we were able to employ the authority of the FOIA to gather timely information, our varied responses highlight the challenge of relying on FOI requests as a highly reliable, reproducible data set and also the need to formulate questions and interpret findings with caution.

Response rates were high with only one ICB unable to answer the request questions. Given that ICBs are responsible for approving funding of medications to be included in regional formularies, it is unlikely that we have overlooked significant data for any region. However, we appreciate that our study is limited to England and therefore does not include data concerning semaglutide provision in Wales, Scotland or Northern Ireland. The England focus of this work, as well as the reliance on FOI data, are the main limitation. The decision to focus on England in the current study was made pragmatically to focus on ICB commissioning policy. Healthcare spending is devolved to individual nations within the United Kingdom and, as such, this work did not assess any potential variation in Scotland, Wales or Northern Ireland. Whilst there is a legal requirement to ensure accuracy of response to FOI requests, given some of the variation in response to seemingly very similar questions, this is a limitation.

Data collected by Sky News Ltd shows that there are as many people on waiting lists for Tier 3 weight management services as currently under a service. However, this data is limited by the unavailability of data from some ICBs, so with data missing from approximately a quarter of ICBs, the numbers of patients within and waiting for Tier 3 weight management services discussed here is likely to be a significant underestimate.

Of the ICBs who provided a full response to OMC FOI requests, 78% (32/41) currently fund both the prescription of and services able to prescribe semaglutide. However, additionally taking into account ICBs who are restricting access based on additional eligibility criteria (two) or only prescribing to those with the greatest clinical need (three), this drops to 66% (27/41).

The restricted eligibility for semaglutide to those with the most clinical need is perhaps surprising given there has been no national directive guiding this from NHS England.

Our study finds that 18 months after the publication of NICE TA875, 22% (7/41) of ICBs do not fund semaglutide and two further restrict access, despite the NHS constitutional requirement to provide access. Furthermore, six of the seven non‐funding ICBs were unable to guarantee that their patient population will have access to semaglutide within 2 years of the initial NICE TA875 release.

This work has identified only 838 patients receiving an NHS prescription of Wegovy from specialist services by April 2024. Whist we acknowledge there is a risk of incomplete data, this is a significant difference from the 13,500 patients that NICE had projected would be on the drug by the end of March 2024 (from authors records; NICE report with this estimate is no longer available). Accurate numbers of patients on private prescriptions are not available.

This may not be surprising given that an audit of ICB 5 year forward plans revealed that only five of 42 ICBs included obesity, or the importance of a healthy weight, as a priority target and 2 ICBs had no mention of obesity in their plans [16]. Further, data on obesity rates was only included in 40% of plans, making it challenging to see how any plans to reduce rates of obesity could have a measurable target.

Determining the barriers to current or planned semaglutide access was beyond the scope of this study and not easily established using our methodology. However, we add to the current literature highlighting the inequity of access to specialist management for overweight and obesity. At the time of this research, there were more patients on a waiting list than receiving care in specialist weight management services. Within services, despite ICBs reporting widespread commissioning of both services and medication, only a very small cohort of patients had received semaglutide as a treatment for obesity on an NHS prescription. The commissioning and availability of medications seem to be a significant problem across England and a barrier to effective treatment. Furthermore, the current capacity of specialist services is clearly grossly inadequate to meet demand. The approach to, and treatment of, obesity will need to be radically different from the status quo if treatment is to become more widely available. With the advent of the tirzepatide TA for overweight and obesity, and the imminent requirement for its use in primary care as well as specialist services, there is likely a need for significant investment in wrap‐around services providing supported self‐management or a wholly novel, untested and potentially harmful strategy of obesity medications being used as stand‐alone therapy without wrap‐around provision, more akin to the treatment of other diseases. Significant investment and policy change will be needed to fully realise the potential benefits of these medications at a population health level. Given the considerable geographic variation highlighted in these requests, central coordination of eligibility and treatment strategies is needed to minimise inequality.

## Author Contributions

A.d.B., H.S. and E.M. led the three sets of FOI requests. H.S. and E.M. and their associated employers agreed to share their data sets and contributed to the revision of the manuscript. P.S. provided critical contributions to the manuscript and integration of the data. A.d.B., R.A., B.M.M., S.L.B. and J.P.H.W. along with J.M.H. determined the FOI requests sent by the academic authorship group, with all providing significant contributions to the manuscript, the first draft of which was written by A.d.B., with significant review and editing by J.M.H. and S.W.F. All authors had access to the data. J.M.H. and A.d.B. act as guarantors of the presented data.

## Conflicts of Interest

A.d.B. has no relevant declarations. H.S. is an employee of Sky News Ltd. E.M. is an employee of The BMJ. P.S. has no relevant declarations. Rob Andrews declares being a PI on a Novo Nordisk education grant awarded to the Society for Endocrinology, Barbara M. McGowan declares a research grant from Novo Nordisk, honoraria as a consultant or speaker for Eli Lilly, Amgen, Novo Nordisk, Pfizer, and Johnson & Johnson and is a shareholder of Reset Health. Sarah Le Brocq declares advisory work for Novo Nordisk and Astra Zeneca as well as honoraria for speaking engagements from Novo Nordisk, Eli Lilly, Medscape and Boehringer Ingelheim, John P. H. Wilding declares consultancy/advisory board work for the pharmaceutical industry contracted via the University of Liverpool (no personal payment) for Altimmune, Amgen, AstraZeneca, Boehringer Ingelheim, Cytoki, Lilly, Napp, Novo Nordisk, Menarini, Pfizer, Rhythm Pharmaceuticals, Sanofi, Saniona, Tern, and Shionogi; research grants for clinical trials from AstraZeneca and Novo Nordisk and personal honoraria/lecture fees from AstraZeneca, Boehringer Ingelheim, Medscape, Novo Nordisk and Rhythm. He is past president of the World Obesity Federation, a member of the Association for the Study of Obesity, Diabetes UK, EASD, ADA, Society for Endocrinology and the Rank Prize Funds Nutrition Committee. From 2009 to 2024 he was national lead for the Metabolic and Endocrine Speciality Group of the UK NIHR Clinical Research Network. Stuart W. Flint declares researcher‐led grants from the National Institute for Health Research, the Office of Health Improvement and Disparities, Doncaster Council, the West Yorkshire Combined Authority, and Novo Nordisk and support for attending academic conferences and events from Novo Nordisk, UK Parliament, Welsh Parliament, and Safefood. Jonathan M. Hazlehurst declares advisory work previously for Novo Nordisk as well as honoraria for speaking engagements from Novo Nordisk and Eli Lilly and support for attendance at academic meetings from Novo Nordisk as well as Eli Lilly as well as academic funding from the NIHR.

## Data Availability

The data that support the findings of this study are available from the corresponding author upon reasonable request.
